# Determining bacterial and host contributions to the human salivary metabolome

**DOI:** 10.1080/20002297.2019.1617014

**Published:** 2019-06-04

**Authors:** Alexander Gardner, Harold G. Parkes, Po-Wah So, Guy H. Carpenter

**Affiliations:** a Department of Mucosal and Salivary Biology, Dental Institute, King’s College London, London, UK; b Division of Radiotherapy and Imaging, Institute of Cancer Research, London, UK; c Department of Neuroimaging, Institute of Psychiatry, Psychology and Neuroscience, King’s College London, Maurice Wohl Clinical Neuroscience Institute, London, UK

**Keywords:** NMR spectroscopy, metabolomics, saliva, oral microbiome

## Abstract

**Background:**

Salivary metabolomics is rapidly advancing.

**Aim and methods:**

To determine the extent to which salivary metabolites reflects host or microbial metabolic activity whole-mouth saliva (WMS), parotid saliva (PS) and plasma collected contemporaneously from healthy volunteers were analysed by ^1^H-NMR spectroscopy. Spectra underwent principal component analysis and k-means cluster analysis and metabolite quantification. WMS samples were cultured on both sucrose and peptide-enriched media. Correlation between metabolite concentration and bacterial load was assessed.

**Results:**

WMS contained abundant short-chain fatty acids (SCFAs), which were minimal in PS and plasma. WMS spectral exhibited greater inter-individual variation than those of PS or plasma (6.7 and 3.6 fold, respectively), likely reflecting diversity of microbial metabolomes. WMS bacterial load correlated strongly with SCFA levels. Additional WMS metabolites including amines, amino acids and organic acids were positively correlated with bacterial load. Lactate, urea and citrate appeared to enter WMS via PS and the circulation. Urea correlated inversely with WMS bacterial load.

**Conclusions:**

Oral microbiota contribute significantly to the WMS metabolome. Several WMS metabolites (lactate, urea and citrate) are derived from the host circulation. WMS may be particularly useful to aid diagnosis of conditions reflective of dysbiosis. WMS could also complement other gastrointestinal fluids in future metabolomic studies.

## Introduction

Saliva is an emerging fluid in diagnostic science, offering advantages over fluids more widely used for diagnostic purposes. Saliva collection is less invasive than plasma collection, and saliva can be produced ad- libitum unlike urine [1]. To allow the full diagnostic potential of saliva to be reached, much research pertaining to salivary biomarker discovery currently emphasises optimisation either of sample handling or analytic techniques [2–6] and assessing physiological modifiers in health such as diurnal variation, diet and age [7,8].

Recently, metabolic profiling of saliva by proton Nuclear Magnetic Resonance spectroscopy (^1^H-NMR) has revealed discriminatory metabolic signatures for several diseases. These include oral pathologies such as dental caries [9,10], periodontal disease [11,12] and Sjögren’s syndrome [13] as well as extra-oral conditions such as dementia [14] and type 1 diabetes [15]. Both caries and periodontal disease are of microbial aetiology. It therefore appears that differences in salivary metabolic composition can reflect dysbiosis of the oral microbiota. Several studies of salivary metabolic composition have touched on the role of bacteria in generating or consuming salivary metabolites [11,16,18]. Despite identification as a subject requiring further research [18], the extent of host-derived and bacterial contributions to salivary metabolites has not specifically been studied. While knowledge of the composition of the oral microbiome has undergone rapid advancements due to advances in sequencing technology, moving towards studying the collective metabolic activity of this complex system, represents a crucial step in understanding oral health and disease [19].

The majority of ^1^H-NMR studies of saliva have been focused on whole-mouth saliva (WMS). WMS is a complex fluid derived from three major paired salivary glands and hundreds of minor glands throughout the oral cavity, containing hundreds of millions of bacteria per millilitre, as well as millions of host-derived cells [4]. The cellular content of WMS has been identified as complicating the analysis of whole mouth saliva, as bacteria and neutrophils may have ongoing metabolic activity, thus care is required in preventing artefactual changes in samples following collection [5].

This issue can be addressed by aseptic collection of saliva directly from the gland. This is most commonly via the parotid gland, where if collected appropriately, the saliva is sterile [20]. This secretion therefore does not enter the oral cavity and is prevented from modification by oral bacteria, reflecting only host metabolic activity. Relatively, few studies have conducted metabolic analysis of parotid saliva (PS) by ^1^H-NMR. However, one of the earliest NMR investigations of saliva was conducted on PS, as well as saliva isolated from the submandibular/sublingual glands. Despite challenges of low field strength and signal to noise ratio, several assignments were made although the aromatic portion of the spectra was not reported [21]. More recently, PS was analysed alongside WMS collected from the same individual, with several differences in the metabolic profile being detected, although the sample size of only one precluded statistical analysis [22].

Analysis of different fluids (typically plasma and urine samples) has been conducted in various studies [23–26], however, inclusion of saliva is rare. One large scale ^1^H-NMR metabolomic study has included saliva alongside plasma and urine, albeit collected at different times, however, the intention was not to draw comparison between the different fluids but simply assess them independently [27].

The aim of the present study was to investigate the extent to which metabolites present in WMS are derived from the oral microbiota or the host by comparison of WMS, PS and plasma collected contemporaneously.

## Materials and methods

### Sample collection

Samples were collected following ethical approval from King’s College London ethics committee (HR-15/ 15-2508) and with the informed consent of participants. A total of 11 healthy (self-reported absence of disease/ill- health), non-smoking volunteers aged 22-31 years with no obvious oral pathology or antibiotic use over the preceding 3 months participated. Participants selfreported oral health status was confirmed by a visual inspection from a qualified dentist, determined by lack of caries, plaque deposition, or gingival inflammation. Radiographic examination was not conducted. Samples were collected during afternoons at least 1 h after oral exposure to exogenous substances (eating, chewing gum, smoking or oral hygiene) or exercise following our previous protocol [4].

Unstimulated WMS was collected by expectoration into sterilised universal tubes, over a period of 5 min. Simultaneously, blood was collected into heparinised capillaries by lancing the finger with a sterile lancet following disinfection with isopropanol. PS was subsequently collected by placing a sterilised Lashley cup over the opening of the parotid duct. The Lashley cup was left in place in the absence of stimulation for 10 min, and unstimulated PS was collected. The cup was then flushed with air, replaced and stimulated PS was collected. Citrate had previously been identified as an endogenous metabolite in PS. To avoid the potential for contamination, 1 ml of 2% food grade tartaric (rather than citric) acid was placed onto the tongue in approximately 0.25 ml increments at 30 s intervals as a stimulus.

### Sample preparation and storage

All fluids were maintained chilled during processing. Saliva samples were centrifuged at 15,000 g for 10 min at 4°C. The supernatant was collected, and the cell pellet was discarded. Although acellular, PS was subject to the same centrifugation as WMS for purposes of standardisation. Blood capillaries were sealed with capillary clay and centrifuged (1,600 g for 15 min at 4°C) to yield plasma as described by Dona et al. [28]. The remaining cell portion was discarded. Samples were centrifuged within minutes of collection, with the exception of a WMS aliquot taken for bacterial culture. Processed samples were then immediately transferred to freezer storage at −80°C prior to analysis.

### Bacterial culture of WMS

Two types of media were prepared. Tryptone, yeast extract and cysteine (TYC) media enriched with sucrose and designed to select for saccharolytic species made to the specification of Wade et al. [29]. The second medium was fastidious anaerobe agar (FAA) with 5% defibrinated horse blood, intended to select for bacteria that catabolise peptides and amino-acids. Prior to centrifugation for NMR (see below), an aliquot of WMS was taken and serially diluted tenfold in sterile phosphate-buffered saline (PBS). Plates of both media types were inoculated with 25 μl of WMS dilution (1:100 for the sucrose media and 1:10,000 for the FAA with horse blood). Plates were grown in an anaerobic cabinet for 48 h, and the colony-forming units per millilitre (CFU/ml) of WMS grown on both media types were estimated.

### ^1^H-NMR spectroscopy

Sample preparation, spectral acquisition, and spectral processing are described in detail elsewhere [4]. Samples were analysed in a 600 MHz spectrometer (Bruker, Karlsruhe, Germany) at a proton frequency of 600.2 MHz at 25°C using a CPMG spin-echo pulse sequence with presaturation to edit out macromolecule resonances from the spectra. The total echo time was 64 min with a relaxation delay of 4 s, and acquisition time of 2.32 s. In total, 256 transients were collected following four dummy scans, with 64 k data points and spectral width of 20 ppm (-5 to 15 ppm). Samples were analysed after a single freeze thaw cycle and maintained at 4°C prior to analysis.

WMS and stimulated PS were prepared unmodified in a 5 mm OD NMR tube (500 μl sample) with a coaxial 3 mm OD NMR tube containing 300 μl 1 mM trimethylsilyl-[2,2,3,3,-^2^H_4_]-propionate (TSP) standard with 50% deuterium oxide. Due to plasma collection by finger prick and the naturally low flow rate of unstimulated PS and plasma, the lower sample volumes of these fluids required different preparation. Unstimulated PS was analysed by putting the sample in the 3 mm OD NMR tube and the standard in the 5 mm OD tube. In most cases, plasma samples were limited to 100 μl, therefore samples were prepared in a 1:2 plasma: buffer ratio as described by Beckonert et al. [30] and analysed in a 3 mm NMR tube. The same protocol recommends formate as an internal standard as opposed to TSP, however, endogenous formate was observed in preliminary plasma samples. Pyrazine was therefore chosen as an internal standard at a concentration of 0.33 mM [31,32]. The pH was adjusted to 7.4. A spectrum of the tartaric acid stimulus was also acquired to ensure there was no contamination of stimulated PS.

### Urea validation

Urea was observed in the biofluid ^1^H-NMR spectra, however, due to proton exchange between urea and water, absolute quantification of urea by ^1^H-NMR was not possible from the acquired spectra [33]. Thus, urea was also measured by a colorimetric assay (Invitrogen). Samples were diluted in water 1:5 for saliva and 1:10 for plasma.

### Statistical analysis

Sample size was estimated *a priori* based on preliminary analysis of previously collected samples and adequate power confirmed for the relevant statistical analyses using G*Power (Universität Düsseldorf, Germany). Data are shown in supplemental Table 2. In addition to manual integration of metabolite peaks as previously described [4], processed spectra were integrated in 0.04 ppm buckets from 0.72 to 8.48 ppm (MestRec v, MestreLab Research). Residual isopropanol signals from the disinfectant swab were detected in some plasma samples, thus buckets corresponding to these regions were excluded from all spectra, as was the water peak (4.5-5.5 ppm). Each bucket integral was normalised to the total spectrum integral thereby removing effects from inter-individual differences in metabolite concentrations.

Principal components analysis (PCA) and k-means cluster analysis were performed on bucketed spectral integrals of all samples in the KNIME analytics platform (Konstanz, Germany). PCA was also conducted to reveal the most discriminatory bucket of spectral regions between WMS and PS. To quantify the spectral variability of the different fluids, the mean Euclidean distance between all samples for each biofluid type was calculated. The first three dimensions of the projected PCA coordinates for each sample were multiplied by the relevant principal componentweighting factor. Euclidean distance between all weighted projected samples of each biofluid type was measured in a pairwise manner (i.e. 55 total measurements for 11 samples) and averaged.

Key metabolite peaks were quantified, inspected for normality and differences were analysed by paired t-test or repeated measures ANOVA with Bonferroni post-hoc where appropriate in SPSS. Metabolite concentrations in WMS were correlated with bacterial load (CFU/ml).

## Results

### Participant and sample details

Samples were collected from 11 participants (5 males, 6 females) aged 22-31, mean age was 25.5 years. All participants had a healthy unstimulated WMS flow rate (> 0.4 g/min). Three participants failed to produce PS in the absence of stimulation. Stimulated PS was successfully collected from all participants.

### Biofluid spectral profile

**Figure 1:**
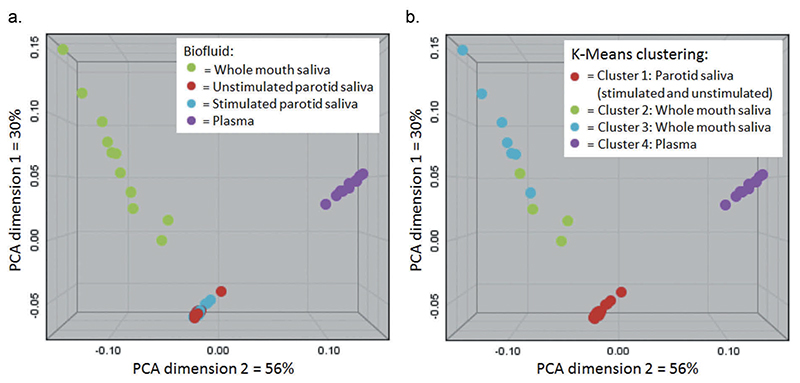
a - PCA plot of all samples, with fluid type indicated by shape. b - K-means cluster analysis of samples with shapes indicating separate statistical clusters. Samples clustered based on fluid type with WMS samples spreading over two clusters and PS and plasma samples forming tighter distinct clusters. Both stimulated and unstimulated PS samples were clustered together. There was no significant biological difference between the WMS samples in clusters 2 and 3.

PCA and K-means cluster analysis of the different biofluid spectra are shown in Figure 1. Spectral profiles of plasma and PS (both stimulated and unstimulated) were observed to individually cluster tightly, whereas WMS spectral profiles were more dispersed and spread over two statistical clusters. There was no overlap of different fluids within any cluster, indicating distinct metabolomic profiles of the different fluids. Unstimulated parotid plasma samples displayed the lowest mean Euclidean distance (i.e. tightest clustering) of the different biofluids. Stimulated PS samples showed slightly wider clustering than unstimulated PS (1.19-fold greater mean Euclidean distance). Relative to unstimulated PS, the mean Euclidean distances between samples for plasma and whole-mouth saliva were 1.87 and 6.74, respectively. The metabolite profile of whole mouth saliva therefore displayed the greatest inter-individual variation of the fluids.

### Individual metabolite concentrations

**Figure 2:**
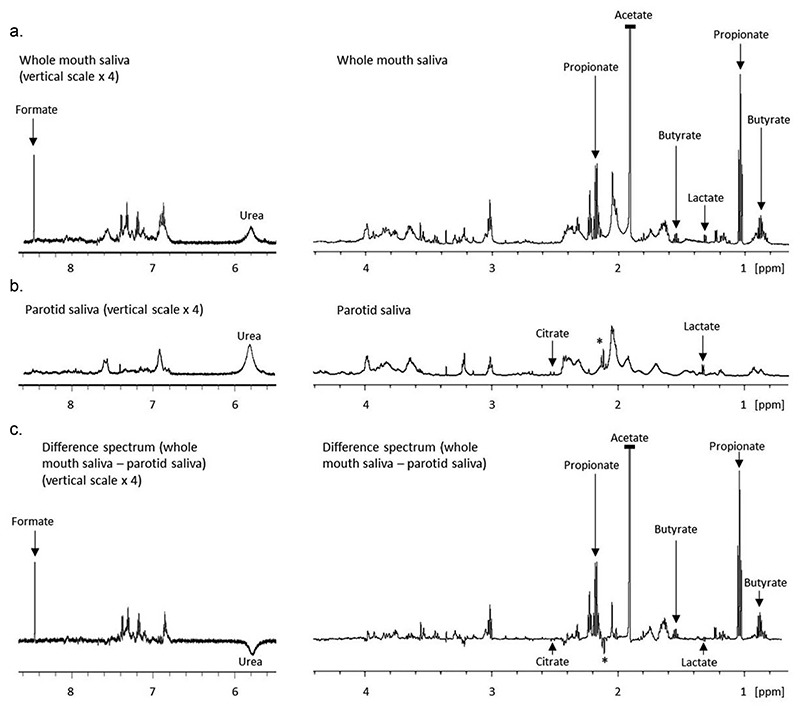
Partial 1D 600 MHz ^1^H-NMR spectra of WMS (a), stimulated PS (b) and the subsequent difference spectrum (WMS minus PS), (c). The aromatic region (8.5-5.5 ppm) is at an increased (x 8) vertical scale relative to the aliphatic region (4.5-0.7 ppm). Peaks marked ‘*’ are unassigned. Acetate has been truncated in the WMS and difference spectra.

A comparison of the spectral profiles of WMS and stimulated PS with key metabolites identified is illustrated in Figure 2. Comparisons are made between WMS and stimulated PS as opposed to unstimulated PS as unstimulated PS is impractical to collect in a clinical setting, with three of our participants failing to produce any of the fluid. No trace of the tartaric acid stimulus (supplemental Figure 1) was identified in stimulated parotid samples, indicating no contamination between sample and stimulus. Figure 1 indicates that stimulation does not affect the overall normalised spectral profile of PS. Individual metabolite concentration of stimulated and unstimulated PS tended not to be significantly different, with the exception of urea, which was higher in unstimulated PS (supplemental Table 1).

The difference spectrum (Figure 2(c)) is a digital subtraction of the PS spectra from the WMS spectra. The spectral profile is similar to that of WMS but with fewer broad peaks and a smoother baseline, likely due to similarities in the macromolecule profile of WMS and PS. Notably lactate and citrate are barely visible in the difference spectra as they are at similar concentrations in both fluids. Alongside urea, the unassigned PS peaks visible at 2.12 to 2.14 ppm in PS (marked ‘*’) are some of the only peaks to be projected negatively, i.e. more concentrated in PS than WMS.

**Figure 3:**
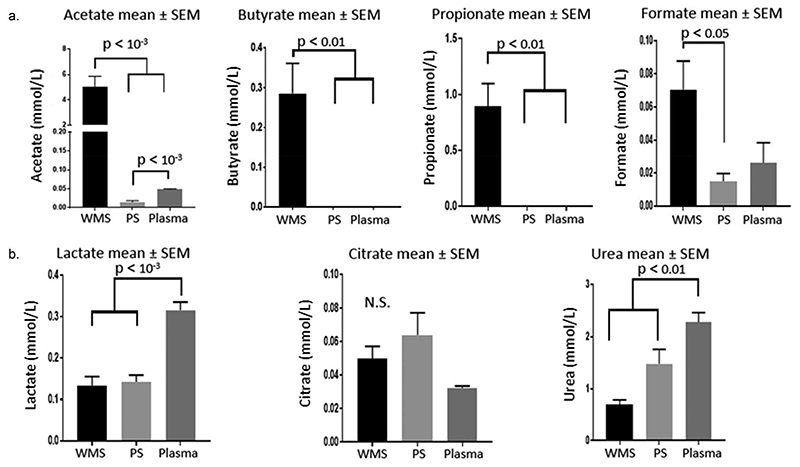
a - Concentrations of short chain fatty acids in WMS, stimulated PS and plasma. For purposes of scale, the y-axis is interrupted for acetate. b – Concentrations of lactate, citrate and urea in WMS, PS and plasma. Urea measurements are from colorimetric assay. All p-values reflect a Bonferroni post-hoc test after repeated measures ANOVA, n = 11.

PCA of WMS and PS spectral regions revealed that the most discriminatory buckets were between 1.88 and 1.96 ppm, likely reflecting the presence of acetate. In WMS, acetate was consistently the most abundant metabolite present but close to the limit of quantification in PS. Other short-chain fatty acids (SCFAs) were similarly abundant in WMS but absent from or sub-quantifiable in PS and plasma including butyrate and propionate. Formate was present in all fluids but was statistically lower in PS than WMS. Concentrations of SCFAs in the biofluids are presented in Figure 3(a).

Other metabolites observed in all fluids included lactate, urea and citrate, Figure 3(b). Lactate levels were significantly higher in plasma than WMS or PS whereas citrate levels were generally higher in PS than plasma although statistical difference was not reached. Urea was also higher in plasma than WMS or PS, with urea generally higher in the PS than WMS.

### Relationship between metabolite concentration and bacterial load

Salivary bacterial load ranged from 500 to 250,000 CFU/ml for saccharolytic bacteria and 9.5 × 10^6^ and 7.8 × 10^8^ CFU/ml for proteolytic bacteria. The relationship between bacterial load and metabolite concentration is presented in Table 1. A number of strong correlations between metabolite concentration and bacterial load were observed, particularly bacterial load of proteolytic bacteria. Notably, these were for the SCFAs, acetate and propionate (the first and second most abundant salivary metabolite, respectively); the amino acids, phenylalanine and glycine; and the organic acids, succinate and pyruvate. Urea was the only metabolite to inversely correlate with bacterial load.

### Relationship between salivary urea measured colorimetrically and by ^1^H-NMR

**Figure 4:**
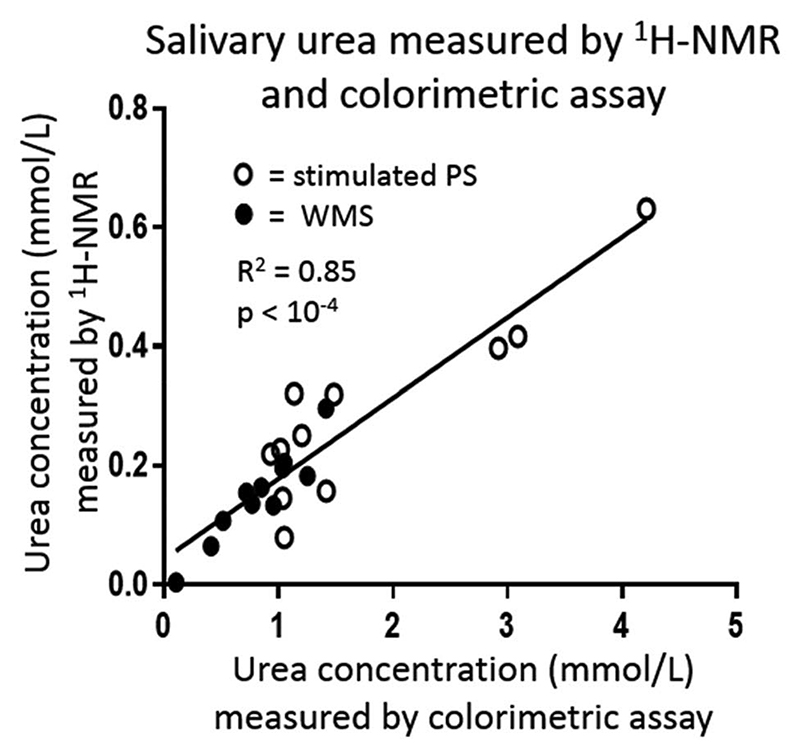
A strong correlation between relative salivary urea concentration measured by ^1^H-NMR and colorimetric assay was observed.

Strong correlations were observed between relative urea concentrations measured by colorimetric assay and by ^1^H-NMR for both WMS and PS (R^2^ = 0.85; p < 10^-4^), Figure 4. However, absolute concentrations measured by ^1^H-NMR were typically five times lower than those measured by colorimetric assay, likely reflecting proton exchange with water and suppression resulting from presaturation of the nearby water signal. For this reason, where absolute concentrations of urea are required rather than relative concentrations, then measurement of urea in biofluids by ^1^H-NMR alone is not ideal.

## Discussion

The metabolic composition of WMS measured by ^1^H-NMR is dominated by SCFAs, in particular acetate, propionate, butyrate, and to a lesser extent, formate. Interactions between gut microbiota (and by extension its metabolome) and host health is a rapidly growing research area [34], with the concept of the metabolome providing a ‘functional readout’ of the microbiome being demonstrated [35]. Acetate, propionate and butyrate are recognised as products of bacterial fermentation in the gut, where they are increasingly understood to have multiple important roles in health [36,37]. While acetate produced in the gut reaches sufficiently high concentrations to enter the systemic circulation, acetate concentrations in glandular PS were several orders of magnitude lower than corresponding levels in WMS. Similarly, the absence of propionate and butyrate in PS compared with relatively high levels in WMS, as well as the strong correlations with salivary bacterial load indicates these metabolites are generated in the mouth by oral bacteria. Such a relationship was also found for some lower concentration salivary metabolites such as methylamine, dimethylamine and trimethylamine. Signalling via tri- methylamine/trimethylamine oxide represents an additional communication pathway between host and microbiota [38]. Given the growing interest in gut microbiota, this work raises the question as to whether there is a link between salivary metabolite profiles and metabolite profile of other gastrointestinal fluids. This has not been directly studied, however, evidence suggests both oral and gut microbiomes relate to cardiovascular health and plasma lipid metabolism [39]. Similarly, whether salivary metabolites have a role in maintaining the epithelial integrity of the oral mucosa analogous to gut metabolites such as butyrate for colonic epithelium remains to be investigated. WMS may present an easily acquired fluid to complement other gastrointestinal fluids in future studies.

Host-derived metabolites appearing to enter WMS from the circulation via the salivary glands are also important constituents of saliva. This study showed no difference between lactate concentration in PS and WMS although salivary lactate had been suggested to be of microbial origin in WMS [18]. Previous work has shown that within seconds of exposure to exogenous sucrose, WMS lactate concentrations experience a rapid increase, as do levels of pyruvate and succinate [4]. The fasted state (1 h) of the participants in this study indicates that in the absence of recent nutrition, salivary lactate is not a significant metabolite of the healthy oral microbiota. The relatively low lactate levels observed in WMS likely reflect a baseline lactate entering PS from circulating plasma. This may explain the lack of correlation observed between lactate (the primary product of saccharolytic oral bacteria such as *Streptococcus mutans)* and the saccharolytic bacterial load. Modifiers of plasma lactate, including exercise, would therefore require consideration when measuring salivary lactate for diagnostic purposes. Differential metabolic activity based on nutrient availability could present a key difference between the metabolome of the oral cavity and the gut, the latter receiving a more constant nutrient presence than the mouth.

The reduction of urea in WMS relative to PS and the inverse correlation with the saccharolytic bacterial load indicates utilisation of host-derived metabolites by the oral microbiota. Urea is implicated in elevating oral pH via conversion to ammonia, thus microbial urea consumption could be a survival mechanism in the presence of increased growth of acidogenic saccharolytic bacteria [40]. In the case of citrate, salivary levels were on average elevated above plasma levels indicating the possibility of either citrate production by salivary glands or active transport from plasma. While it is well-recognised that fluids such as prostatic fluid contain high levels of actively concentrated citrate [41], the role of salivary citrate is unclear. Given the fact that citrate concentrations are maintained to a greater degree than the total protein concentration (supplemental Table 1) during stimulation of PS secretion, whereby the fluid output increases at least ten-fold [42], this suggests an active transport mechanism of citrate into PS.

This study suggests that the metabolic composition of saliva is more reflective of the microbiota than the underlying host metabolism. As evidenced by PCA of ^1^H-NMR spectra of WMS, PS and plasma, the basic metabolic fingerprint of WMS displays much greater inter-individual variation than that of PS and plasma. This likely reflects the diversity of the oral microbiota modulating WMS metabolites, whereas PS and plasma are largely influenced by host physiology and therefore more closely regulated. Compared to WMS, literature on PS as a diagnostic fluid is lacking, however increased study of this fluid either alone or alongside whole mouth saliva could open new avenues of diagnostic information. Given the growth of research into hostmicrobiome interactions, insight into the net microbial activity in the oral cavity afforded by salivary metabolic profiling represents an additional advantage of saliva as a diagnostic fluid. This is particularly true considering multiple oral diseases are of bacterial aetiology [9,11].

## Supplementary Material

Supplemental material

## Figures and Tables

**Table 1:** Summary of whole mouth saliva metabolite concentration and their correlation with salivary bacterial load. (* = p < 0.05; ** = p < 0.01), n = 11. Urea measurements are from colorimetric assay

Molecule class	Metabolite	Concentration (mmol/L) Mean (Range)	Correlation with sucrose enriched media (saccharolytic bacterial load) (Pearson’s r; p value)	Correlation with horse blood/peptone enriched media (proteolytic bacterial load) (Pearson’s r; p value)
Short chain fatty acids	Acetate	5.03 (1.53–12.26)	0.72*;0.01	0.85**;0.001
	Butyrate	0.29 (0.12–1.00)	0.72*;0.01	0.70*;0.02
	Propionate	0.90 (0.12–2.60)	0.67*;0.02	0.83**;0.002
	Formate	0.07 (0.006–0.13)	NS	NS
Amines	Dimethylamine	0.01 (0.003–0.015)	0.63*;0.04	0.64*;0.04
	Methylamine	0.012 (0.008–0.021)	NS	0.71*;0.01
	Trimethylamine	0.007 (0.002–0.015)	0.74**;0.009	0.67*;0.02
Amino acids	Phenylalanine	0.06 (0.02–0.16)	0.74**;0.009	0.80**;0.003
	Glycine	0.26 (0.06–1.07)	0.78**;0.004	0.82**;0.002
	Tyrosine	0.10 (0.04–0.18)	NS	NS
	Histidine	0.06 (0.02–0.08)	NS	NS
	Alanine	0.08 (0.013–0.12)	NS	NS
Organic acids	Citrate	0.05 (0.02–0.10)	NS	NS
	Lactate	0.13 (0.05–0.27)	NS	NS
	Pyruvate	0.19 (0.10–0.39)	NS	0.83**;0.001
	Succinate	0.24 (0.10–0.61)	NS	0.81**;0.003
Other	Choline	0.03 (0.10–0.06)	NS	0.72*;0.012
	Methanol	0.05 (0.02–0.10)	NS	NS
	Acetoin	0.04 (0.02–0.08)	NS	NS
	Taurine	0.19 (0.05–0.25)	NS	NS
Amides	Urea	0.83 (0.11–1.41)	– 0.81**;0.003	– 0.63*;0.04
