# Supplementary material for: Determining bacterial and host contributions to the human salivary metabolome
Source: J Oral Microbiol. 2019 Jun 4;11(1):1617014. doi: 10.1080/20002297.2019.1617014 (PMC7610937; doi:10.1080/20002297.2019.1617014)
Supplement: Supplemental Material [file ZJOM_A_1617014_SM8647.docx]

**Bacterial and host contributions to the human salivary metabolome**

**Supplemental Material**

**
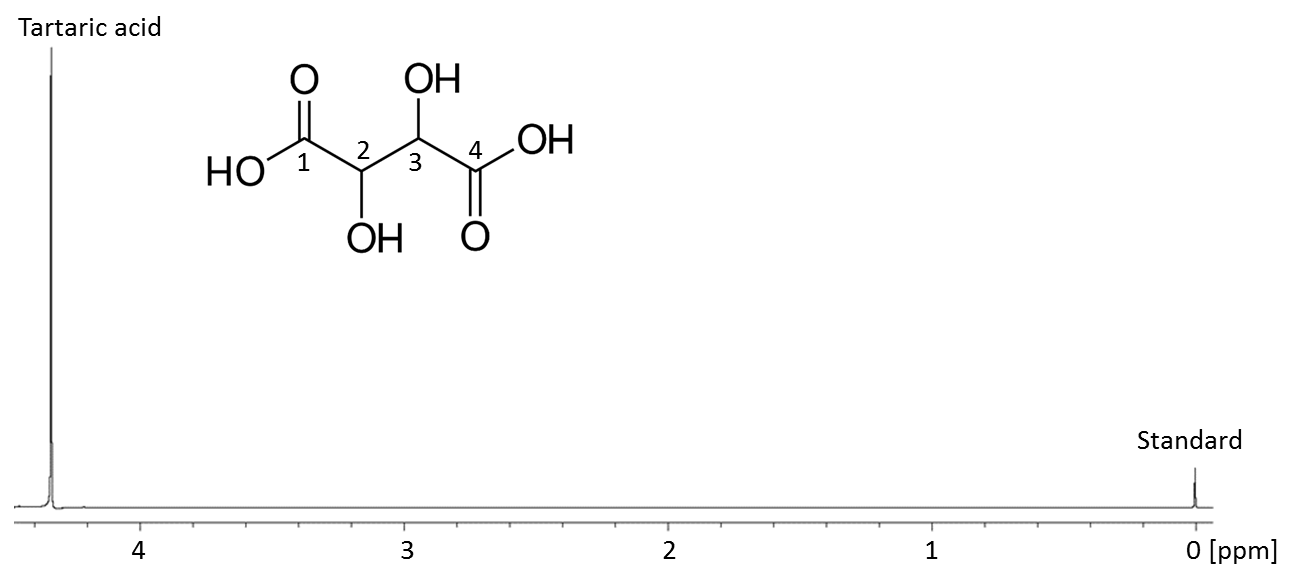
**Supplemental Figure 1: Partial 1D 600 MHz spectrum of tartaric acid, showing a singlet at 4.33 ppm arising from the equivalent protons on carbons 2 and 3. No peak at this region was observed in stimulated parotid saliva samples, indicating no contamination between sample and stimulus occurred.

Supplemental Table 1: Comparison of metabolite and protein concentration in stimulated and unstimulated parotid saliva, (n=8). Protein was measured using a bicinchoninic acid assay (Thermo-Scientific, Rockford, Illinois, USA.) Unassigned metabolite concentration is expressed in relative units as the concentration cannot be determined without knowing the structure. Except citrate mean concentration of all analytes decreased upon stimulation, however this was significant (p<0.05) only for urea and protein.

| **Analyte** | **Unit** | **Unstimulated mean ± SEM** | **Stimulated mean ± SEM** | **P-value (paired t-test; * = p < 0.05)** |
| --- | --- | --- | --- | --- |
| Citrate | mmol/L | 0.03 ± 0.01 | 0.03 ± 0.01 | 0.98 |
| Lactate | mmol/L | 0.10 ± 0.02 | 0.04 ± 0.01 | 0.07 |
| Urea | mmol/L | 2.70 ± 0.22 | 1.74 ± 0.42 | 0.02* |
| Unassigned | NA | 0.24 ± 0.05 | 0.15 ± 0.04 | 0.30 |
| Total protein | mg/dl | 316 ± 49 | 152 ± 18 | 0.02* |

Supplemental Table 2: For metabolites where statistical differences were detected by ANOVA adequate power was confirmed. Power calculations for a repeated measures ANOVA with three multiple comparisons, with an alpha level of 0.05 were conducted. A threshold of power > 0.8 was deemed sufficient to accept the result of the ANOVA, therefore the power to accept the ANOVA result for formate is slightly below threshold.

| **Metabolite** | **Power** |
| --- | --- |
| Lactate | 0.97 |
| Propionate | 0.92 |
| Formate | 0.66 |
| Acetate | 0.96 |
| Butyrate | 0.92 |
| Urea | 0.96 |
